# Using machine learning to speed up manual image annotation: application to a 3D imaging protocol for measuring single cell gene expression in the developing *C. elegans* embryo

**DOI:** 10.1186/1471-2105-11-84

**Published:** 2010-02-11

**Authors:** Zafer Aydin, John I Murray, Robert H Waterston, William S Noble

**Affiliations:** 1 Department of Genome Sciences, University of Washington, Seattle, WA 98195, USA; 2 Department of Computer Science and Engineering, University of Washington, Seattle, WA 98195, USA

## Abstract

**Background:**

Image analysis is an essential component in many biological experiments that study gene expression, cell cycle progression, and protein localization. A protocol for tracking the expression of individual *C. elegans *genes was developed that collects image samples of a developing embryo by 3-D time lapse microscopy. In this protocol, a program called StarryNite performs the automatic recognition of fluorescently labeled cells and traces their lineage. However, due to the amount of noise present in the data and due to the challenges introduced by increasing number of cells in later stages of development, this program is not error free. In the current version, the error correction (*i.e*., editing) is performed manually using a graphical interface tool named AceTree, which is specifically developed for this task. For a single experiment, this manual annotation task takes several hours.

**Results:**

In this paper, we reduce the time required to correct errors made by StarryNite. We target one of the most frequent error types (movements annotated as divisions) and train a support vector machine (SVM) classifier to decide whether a division call made by StarryNite is correct or not. We show, via cross-validation experiments on several benchmark data sets, that the SVM successfully identifies this type of error significantly. A new version of StarryNite that includes the trained SVM classifier is available at http://starrynite.sourceforge.net.

**Conclusions:**

We demonstrate the utility of a machine learning approach to error annotation for StarryNite. In the process, we also provide some general methodologies for developing and validating a classifier with respect to a given pattern recognition task.

## Background

Recent advances in microscopy, fluorescent tagging and automated image analysis have led to the development of high-throughput methods for monitoring gene expression at single-cell resolution over time [1-4].

**Figure 1:**
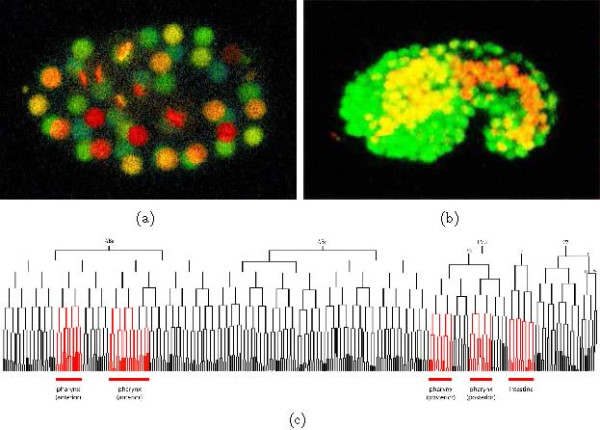
***C.elegans* cell lineaging images**. (a) One stack of images taken during development, with the z-axis represented as a red-green-blue color scale. (b) One image, with two channels corresponding to ubiquitous expression (green) and expression via the pha-4 promoter (red). (c) The worm lineage of 959 cells, with branch lengths determined by StarryNite. Red colored branches indicate expression from the pha-4 promoter.

We focus in this work on a particular protocol for tracking the expression of individual *C. elegans *genes during embryonic development. Briefly, the protocol works as follows. First, histones are genetically tagged with green fluorescent protein (GFP). During development, a stack of confocal microscopy images of the embryo is taken every 50-90 seconds. Each stack contains 31-35 image planes with a spatial resolution of 1 micron. A sample image stack is shown in Figure 1(a), where the z-axis is coded using a red-green-blue color scale (*i.e*., red is the closest and blue is the farthest from the top plane). In this figure, nuclei of nondividing cells are spherical, and nuclei of dividing cells have elongated shapes. Typically, the diameter of a cell nucleus is from 3-11 microns; therefore, each nucleus is represented in 4-11 image planes. A typical image series might track development over 150-200 time points until the embryo consists of 180 cells. With 30 images per time point, such a series consists of 5000-6000 images. These images show approximately 400 cell divisions and 10,000 nuclei at different time points. If we continue to the 350 cell stage, then the series contains 180-230 time points, approximately 700 cell divisions and 20,000 nuclei. In addition to tagging all nuclei using a ubiquitously expressed histone, the protocol can be extended to trace the expression of individual genes. In Figure 1(b), a histone is tagged with GFP, and a second histone is tagged with RFP, where the RFP histone's expression is driven by the promoter associated with the gene pha-4. Hence, the pattern of red nuclei shows in which cells the pha-4 promoter is active.

Once the image samples are collected, the primary analysis task is to identify individual nuclei and to trace the lineage of the individual cells. Because the lineage is highly stereotyped, this task amounts to mapping the observed data onto a lineage tree with fixed topology and variable branch lengths. Such a tree is shown in Figure 1(c) for a two-channel experiment, where the lineage is colored according to whether the monitored gene is expressed along a given lineage. The software tool StarryNite was developed specifically to solve this cell lineaging task [5]. StarryNite can trace a 350-cell stage image series in approximately 25 minutes on a desktop computer. However, annotation with StarryNite must typically be followed by a manual curation step, because the automatic annotation contains errors. This curation generally takes approximately two hours to edit a lineage up to the 194-cell stage and four hours to the 350-cell stage [6]. In this work, our goal is to use machine learning methods to reduce this manual annotation time. Using a collection of manual annotations, we systematically analyze the types of errors made by StarryNite. For the most common type of error, we then design a collection of features that encode relevant information about the source of the errors. Finally, we use these features, in conjunction with labels derived from manual annotation, to train a support vector machine (SVM) classifier to identify StarryNite errors with high accuracy. The resulting classifier significantly speeds up the time required to manually curate expression image series. The classifier is built into the latest version of StarryNite http://starrynite.sourceforge.net.

## Results

### Analyzing StarryNite errors

Initially, we investigated the types of errors produced by StarryNite, with the goal of focusing our analyses on the most common errors. To this end, we grouped StarryNite errors into five categories: (1) false positives, (2) false negatives, (3) positioning errors, (4) incorrect diameter estimation and (5) tracing errors. A false positive occurs when StarryNite mistakenly detects a nucleus, which in fact is non-existent. Conversely, false negatives are nuclei that StarryNite fails to identify. Positioning errors occur when StarryNite makes mistakes in finding the coordinates of the centroid of the nucleus. Incorrect size estimation happens when the inferred diameter of a nucleus differs from the true value. Tracing errors include cases where a nucleus at a particular time point is not matched to the right nucleus (or nuclei) in the next time point. For each nucleus, there can be three possible matches: one to one, one to two, or one to none, corresponding to movement, cell division (*i.e*., division call), and cell death [5]. A moving nucleus simply changes its location from one time point to the next. A dividing nucleus splits into two children nuclei in the next time point. Finally, a cell death corresponds to the case where a cell disappears. Once the embryo finishes its development it starts to crawl away from the imaging foci. Hence, in the final stages of development, some cells will start to disappear from the image data and some will still be present. Note that all of these errors are subjectively defined, ultimately, by visual inspection by a human expert. Hence, there is no hard and fast rule for, for example, how far off the centroid must be in order to qualify as a positioning error.

**Figure 2:**
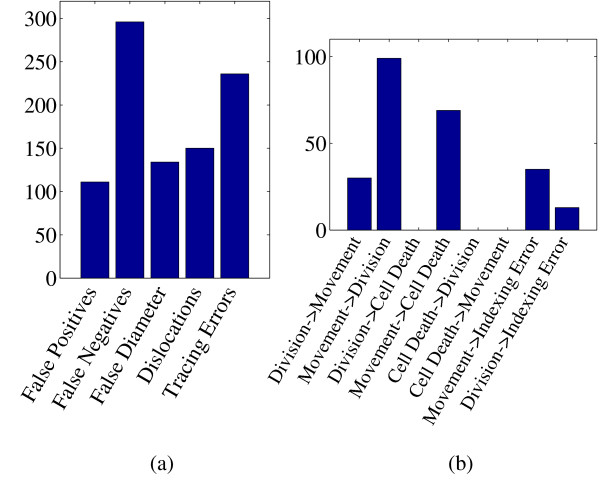
**Histogram of various types of errors in one image series**. (a) Major error types. (b) Subtypes of tracing errors.

We collected statistics for each error type on a single benchmark series (081505), which contains image data up to the 195 cell stage. This series contains a total of 23,987 nuclei annotations by StarryNite and 24,355 annotations in the manually edited version. The results, summarized in Figure 2(a), suggest that false negatives are the most common error types, followed by tracing errors, dislocations, incorrect diameter estimations and false positives. Although false negatives are the most commonly observed errors, we chose to concentrate on the second most common error type, tracing errors. We made this choice for two reasons. First, tracing errors are directly amenable to correction by a simple classifier, which can be applied systematically to all division calls made by StarryNite. In contrast, a classifier that attempts to correct false negative annotations would have to be applied to all empty regions of all image stacks. Second, tracing errors have a more complex morphology than simple false negative annotations, allowing us to use a rich set of features, as described below.

**Figure 3:**
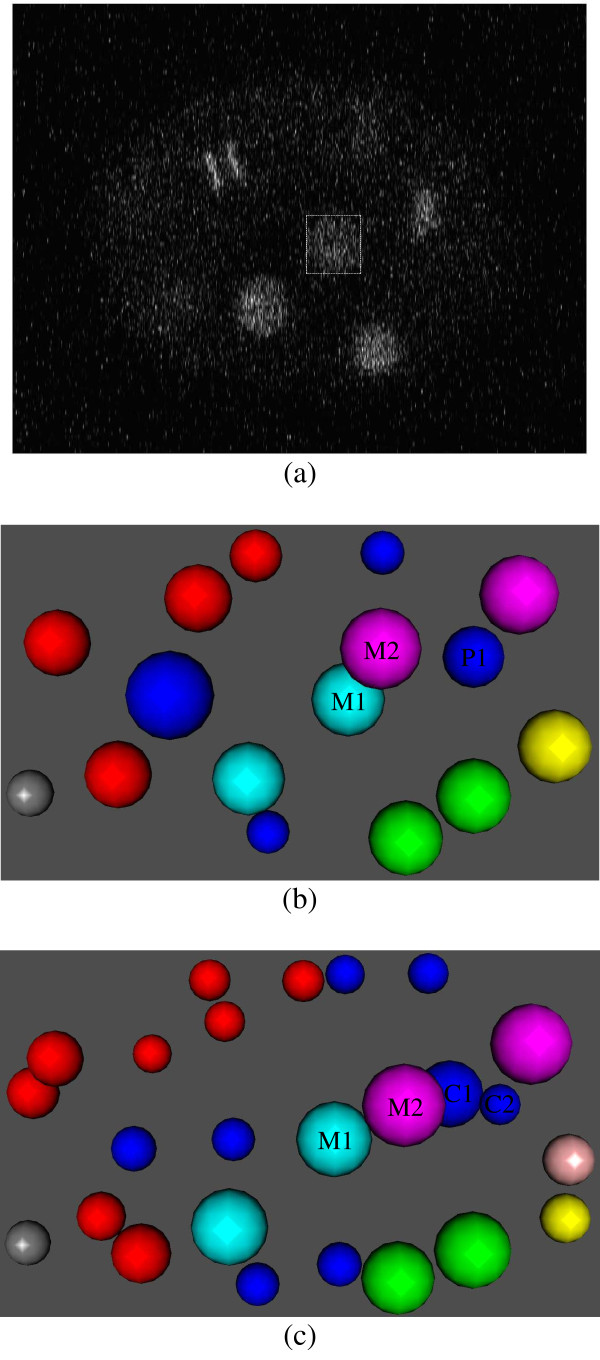
**Moving nucleus annotated as dividing**. (a) An image plane from the series 081505 at *t *= 35 and *z *= 23, where *t *is the time index and *z *is the plane index within the image stack. A moving nucleus is encapsulated by a white square box. (b) 3D view of the nuclei present at *t* = 35. M1 and M2 move from *t* = 35 to *t* = 36 and P1 at *t* = 35 divides into C1 and C2 at *t* = 36. (c) 3D view of the nuclei present at *t* = 36. StarryNite annotates M1 at *t* = 35 as the parent nucleus and links it to M2 and C1 at *t* = 36, which are incorrectly annotated as the children of M1.

Tracing errors can be further subdivided into eight categories: (1) division annotated as movement, (2) movement annotated as division, (3) division annotated as cell death, (4) movement annotated as cell death, (5) cell death annotated as division, (6) cell death annotated as movement, (7) indexing errors of moving nuclei and (8) indexing errors of dividing nuclei. An indexing error of a moving nucleus occurs when a moving nucleus at a particular time point is linked to the wrong nucleus at the next time point. Similarly, an indexing error of dividing nuclei occurs when the indices of the newborn children are incorrectly assigned. Figure 2(b) shows that "movement annotated as division" is the most frequent type of tracing error: 42.3% of the tracing errors in series 081505 are of this type. Indeed, this series contains a total of 427 division calls, and 102 (24%) of those were in fact movements. In addition to being the most frequent tracing error type, movements detected as divisions are biologically important as well. Figure 3(a) illustrates one such error. In the figure, a moving nucleus at a particular time point *t *is encapsulated by a white square box. For simplicity, the figure shows only a single image slice, corresponding to a fixed z-value. Alternatively, Figures 3(b) and 3(c) contain 3D image representations of all the nuclei present at *t *and *t *+ 1, respectively, where *t *= 35 in this example. According to the human annotator, M1 and M2 move from *t *= 35 to *t *= 36 and P1 at *t *= 35 divides into C1 and C2 at *t *= 36. However, StarryNite annotates M1 at *t *= 35 as the parent nucleus and links it to M2 and C1 at *t *= 36, which are incorrectly annotated as the children of M1. Thus, in this example a moving nucleus (M1) is annotated as dividing, causing a deviation from the true topology of the lineage tree. Based on these analyses, we decided to focus our initial efforts on the automatic recognition of movements detected as divisions.

### Feature design

Often, success in machine learning depends critically upon the ability of the researcher to successfully incorporate into the learning framework significant prior knowledge about the problem domain. Such prior knowledge can be represented, for example, using a formal, probabilistic prior or, for kernel methods, by selecting an appropriate similarity function. However, perhaps the most straightforward way to encode prior knowledge is by designing feature extraction routines that are tailored to the task and the data. In our case, we examined the "movement annotated as division" tracing errors in several of our image series and, on that basis, designed a collection of 82 features that provide a rich view of the types of errors produced by StarryNite. The 82 features are summarized in Table 1 and explained below.

**Table 1:** The set of 82 features is grouped into nine categories

	Feature group	Number of features
1	Absolute time index	1
2	Ages	3
3	Diameters	3
4	Distances	15
5	Normalized nucleus support	39
6	Angles	5
7	GFP signals	6
8	Number of nuclei at a given time	1
9	Coordinates	9

	Total	82

#### Time index

The time index denotes how much time has elapsed since the start of embryonic development. The relationship between developmental age and time index depends, of course, on the time resolution of the experiment. In general, StarryNight makes more errors, on a per-nucleus basis, at later time points, simply because the images at later time points contain more nuclei and are hence more crowded.

#### Age

A cell needs to mature to a certain age before dividing into two nuclei. Therefore, by including age information we aim to eliminate incorrect division annotations that correspond to divisions of very young cells or lead to very long-lived cells. We compute ages of the parent nucleus as well as the two children nuclei, as described in Methods.

#### Diameter

We obtain the diameter, in pixels, of the parent and the two children nuclei directly from StarryNite's annotation. We expect the diameters of the children to be similar to one another and smaller than the diameter of the parent.

#### Distance

We include 15 distance features that capture the spatial relationships among the parent, the parent's neighbors and the two children.

#### Normalized nucleus support

During mitosis, a cell typically elongates in one direction, deviating from its usual spherical shape. To enable discriminating between dividing and non-dividing nuclei, we introduce a feature called *normalized nucleus support *that quantifies how spherical the nucleus is. Details are provided in Methods.

#### Angle

During mitotic division, the two children typically move in opposite directions. To capture these directional changes, we define a set of five angle features, as described in Methods.

#### GFP signal

Similar to the diameter features, we expect the GFP signals of the two children to be similar to each other and less than the GFP of the parent. To capture this information, we include six GFP features: the GFP signals of the the parent at *t *- 1, the parent at *t*, children at *t *+ 1 and children at *t *+ 2.

#### Number of nuclei at a given time

This feature allows the learner to exploit the correlation between the number of nuclei at a given time point and the probability of error. When an image stack contains many cells, the nuclei are packed more tightly together and more likely to experience collisions that affect the direction of moving nuclei. Accordingly, we observed that StarryNite makes more errors when the number of nuclei is high.

#### Coordinates

We included the x-y-z coordinates for the centroids of the parent as well as the two children (9 features). With this set of features, we allow the learner to identify a tendency for StarryNite to make more errors at particular locations.

### Preliminary feature analysis

**Figure 4:**
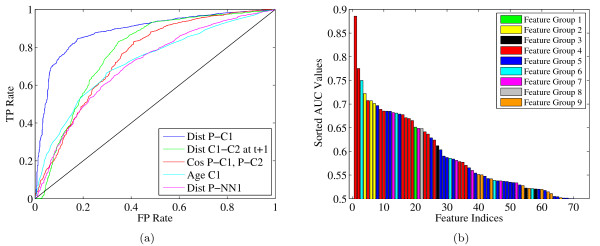
**Analysis of individual features**. (a) The figure plots ROC curves for the features with the top five AUC scores. Feature names are defined as follows: "Dist P-C1" is the distance from parent to child-1; "Dist C1-C2 at t+1" is the distance between children at t+1; "Cos P-C1, P-C2" is the cosine of the angle between (parent to child-1) and (parent to child-2); "Age C1" is the age of child-1; "Dist P-NN1" is the distance from parent to the nearest neighbor of parent. (b) The AUC associated with each feature. The features are sorted according to the AUC.

Prior to performing any machine learning, we measured the discriminative power of each feature individually. The goal of this analysis is two-fold: to identify features that are not individually informative, and to provide a performance baseline against which to compare our machine learning results. Using a development data set consisting of 10 experimental series (see "Benchmark Datasets" for details), we ranked all of the division calls according to each of the 82 features. Each such ranking induces a receiver operating characteristic (ROC) curve, which plots the true positive rate as a function of false positive rate as we traverse the ranked list. We use the area under the curve (AUC) as a performance metric to rank features. Figure 4(a) shows the ROC curves for the top five features, according to this metric, and Figure 4(b) illustrates sorted AUC values across 70 features (only features with non-zero AUC values are shown). In Figure 4(b), the features are sorted by their AUC values. 82 features along with their AUC scores are listed in Additional file 1: features_and_aucs.xls.

This ROC analysis leads to several observations. First, some of the normalized nucleus supports (the ones that are far from the centroid) are zero for all examples in the dataset we used, suggesting that all the nuclei we evaluated are smaller in size than expected. These non-informative features were eliminated from all subsequent analyses. Second, the best feature is "distance from parent to child-1" with an AUC of 0.8857. This provides a baseline against which to compare our trained classifier.

In general, the single-feature rankings are consistent with our biological expectations. For instance, correct divisions have a higher average distance between parent and child-1 than incorrect divisions, because we expect a certain amount of separation between parent and children. When a child candidate is too close to a candidate parent, then StarryNite is more likely to make an incorrect division call linking those close cells. Furthermore, in mitosis we expect the children to move rapidly from each other, resulting in a certain amount of separation between them. This separation is related to the second best feature. We expect the children to move in opposite directions from each other, which can be captured by the third best feauture. We expect the age of a newborn child to be larger than the time it takes a regular moving cell to divide starting from the current time point. This information is represented in the fourth best feature. Finally, we expect a distance relation between a parent and its neighbors, as we observed in the fifth best feature, because if they are close to each other StarryNite is more likely to get confused about choosing the right parent and labeling a moving nucleus as a dividing one.

### Initial testing of an SVM classifier

We performed a 10-fold cross-validation experiment on the development data set. At each cross-validation iteration, we chose one experimental series as the test set and used the remaining series as the training data. Then we learned the optimal parameters and hyperparameters of the SVM classifier by performing internal cross-validation on the training set (see "SVM Classifier"), and we classified each division call in the test set as correct ("Dividing") or incorrect ("Moving").

**Figure 5:**
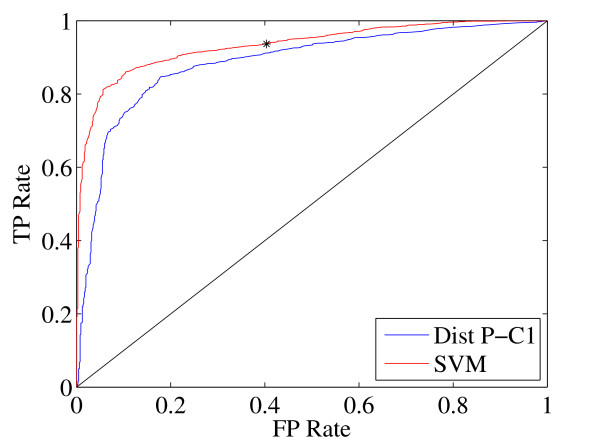
**ROC curves of the best feature and the SVM**. Cross-validated ROC curve produced by the SVM on the development data set and the ROC curve of the best performing single feature ("distance from parent to child-1"). The SVM decision threshold is indicated by an asterisk.

At the threshold selected by the SVM, we achieve an accuracy of 88.16%, which represents a 4.3% improvement over StarryNite (83.84%). Several additional performance metrics are detailed in Table 2. By definition, StarryNite has 100% sensitivity, since we only consider division calls made by StarryNite. On the other hand, our method is 8.5% better than StarryNite in terms of the precision rate (*i.e*., the likelihood of a prediction to be correct) although it annotates some of the true division calls made by StarryNite as errors. We should note that, for guiding manual reannotation, it is better to identify more errors to speed up the editing process even if some of the movement annotations made by the SVM are in fact divisions that are correctly captured by StarryNite. Such incorrect annotations of our method can still be corrected by the human expert, reducing the overall effort that needs to be spent for the editing phase. Figure 5 shows the ROC curve achieved by the SVM, with a point indicating the selected decision threshold. For comparison, we also include the ROC curve produced by the best-performing single feature. The AUC score of the SVM classifer is 0.9330, which is better than the AUC score of the best feature (0.8857). These results show that the SVM classifier is capable of identifying this particular class of StarryNite errors with high accuracy.

**Table 2:** Comparison of the SVM and StarryNite on the development set.

Metric	SVM	StarryNite
True Positives	3178	3393
True Negatives	390	0
False Positives	264	654
False Negatives	215	0
Sensitivity	93.663	100.0
PPV	92.330	83.840
Accuracy	88.164	83.840

Sensitivity/recall is defined as TP/(TP+FN) and positive predictive value (PPV)/precision is computed as TP/(TP+FP)

### Feature selection

Having established a baseline accuracy in the previous experiment, we next explored the possibility of achieving improved performance by eliminating uninformative or redundant features from the classifier. We performed two such experiments, both of which suggest that feature selection for this particular task is not necessary.

**Figure 6:**
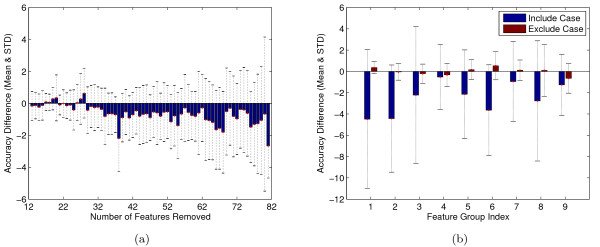
**Two feature selection experiments**. (a) The figure plots the mean difference in accuracy, across 10 cross-validation splits, of an SVM that uses all features compared to an SVM with some features removed. The number of features eliminated is given on the x-axis. Bars above the y-axis represent SVMs that yield better performance than the baseline SVM, and vice versa. Error bars correspond to standard deviations. (b) This figure is similar to panel (a), except that features are considered in groups, as listed in Table 1. Each blue bar compares the accuracy of the 70-feature SVM to an SVM trained from a single feature group, whereas each red bar compares the full SVM to an SVM trained from all feature groups but one.

In the first feature selection experiment, we adopt a simple filter, based on the per-feature AUCs shown in Figure 4(b). Figure 6(a) shows the result of a series of tests conducted with smaller and smaller feature sets. In each step, we eliminated one feature with the lowest AUC. We then performed the same cross-validation experiment described in the previous section, including internal cross-validation to select hyperparameters. For each cross-validation split, we compare the accuracy of the reduced-feature SVM with the accuracy of the baseline SVM that uses all 70 features. The figure shows that, although some reduced feature sets yield a slight improvement in accuracy--e.g., eliminating the worst 28 features gives an improvement of 0.622% --the mean is always less than one standard deviation from zero. This result suggests that this simple feature selection strategy does not significantly improve the performance of the classifier.

In the second feature selection experiment, we considered the joint effect of groups of related features. In this analysis, we used the nine feature groups introduced in the "Feature Design" section. Rather than considering all 2^9 ^- 1 = 511 possible combinations of groups, we considered 18 possibilities: each one of the nine feature groups alone, and all combinations of eight feature groups. As before, we performed a cross-validation experiment on each reduced feature set and then compared the accuracy of the reduced-feature classifier to the accuracy of the baseline SVM. The results shown in Figure 6(b) agree with the previous experiment: in no case does the reduced-feature SVM significantly out-perform the baseline SVM.

Although these two experiments do not prove that feature selection for this particular task is a bad idea, they do suggest that any gains provided by feature selection are likely to be modest. On the basis of these experiments, we therefore decided not to pursue more sophisticated feature selection experiments.

### Evaluation on two validation sets

Finally, we tested the SVM classifier on independent data. Our goal was to find a set of SVM parameters that yield good generalization performance with respect to previously unseen data. In pursuit of this goal, we performed two rounds of analyses, on the two validation sets described in Tables 3 and 4.

**Table 3:** Development and validation sets.

Development set				Validation set			
Experiment	Correct	Incorrect	Total	Experiment	Correct	Incorrect	Total
081305	356	59	415	083005xx	366	36	402
081505	323	83	406	083105	323	47	370
081905	317	105	422	083105xx	325	40	365
081905xx	368	52	420	090105	337	73	410
082005	294	107	401	090205	331	75	406
082205	304	53	357	090205yy	393	31	424
090405	320	91	411	090305xx	353	29	382
090905xx	308	36	344	090605xx	275	50	325
082505	406	37	443	090705	362	34	396
082605	397	31	428	081405	366	30	396

Sum	3393	654	4047	Total	3431	445	3876

For each experimental series, the table lists the total number of division calls made by StarryNite ("Total") as well as the number of those calls that are actually divisions ("Correct") and that are erroneous annotations ("Incorrect")

**Table 4:** The second validation set.

Validation set 2			
Experiment	Correct	Incorrect	Total
20090401_C25D7_10_1_L1	317	99	416
20081007_nhr-68_14_L1	551	176	727
20080524_hnd-1_6_L2	280	153	433
20080731_ceh-32_4_L2	564	313	877
20080929_nhr-67_3_L2	682	315	997
20080524_hnd-1_6_L1	296	160	456
20080912_tbx-37_b12_L1	484	204	688
20080925_ZK185_1_3_L1	673	267	940
20090225_lir-3_10_L1	361	111	472
20080709_ceh-27_1_L1	666	150	816

Sum	4874	1948	6822

For each experimental series, the table lists the total number of division calls made by StarryNite ("Total") as well as the number of those calls that are actually divisions ("Correct") and that are erroneous annotations ("Incorrect")

Initially, we performed a similar cross-validation experiment as before using this new data. The results are shown in Table 5, in the column labeled "CV SVM." Apparently, this data set is easier for StarryNite, which achieves a 4.7% improvement in accuracy, compared to the development data set (88.52% versus 83.84%). However, the SVM still provides a significant boost in performance, giving a 5.8% improvement relative to StarryNite (94.35% versus 88.52%).

**Table 5:** Comparison of SVM and StarryNite on the validation data set.

Metric	CV SVM	Dev SVM	StarryNite
True Positives	3375	3157	3431
True Negatives	282	366	0
False Positives	163	79	445
False Negatives	56	274	0
Sensitivity	98.37	92.01	100.0
PPV	95.39	97.56	88.52
Accuracy	94.35	90.89	88.52

Two sets of SVM results are included: from a collection of SVMs trained via cross-validation on the validation data set ("CV SVM"), and from a single SVM trained on the development set ("Dev SVM")

Unfortunately, when we use the validation data set as a test set--i.e., when we train on the development set and test the resulting SVM on the validation set--our results are not as good. The SVM, using hyperparameters selected on the development set, achieves an accuracy of only 90.9%, which is only 2.4% better than StarryNite's accuracy of 88.5%. This difference is statistically significant according to McNemar's test with a *p*-value of 0.0003. On the other hand, this improvement is smaller than what we achieved via cross-validation on the development set (4.3%) or the validation set (4.7%), suggesting that, although the SVM does a good job of learning to identify errors, those two data sets contain systematic differences that make it difficult for the SVM to generalize from one to the other. We have thus violated the basic premise of most machine learning algorithms, that the test data is drawn from the same underlying distribution as the training data. This hypothesis is supported by the observation that the hyperparameters selected during internal cross-validation are quite different from one another: the learned hyperparameters for the development set were *C *= 66.3692, γ = 2^-11^, and for the validation set *C *= 2.02018, γ = 2^-7^.

As mentioned above, our ultimate goal is to produce a static SVM classifier that yields robust performance across a variety of possible data sets. Because our experiments suggest that our initial development and validation sets contain systematic differences, we next trained an SVM on the combination of the two data sets and tested the performance of the classifier on a second validation data set, which contains ten new series (Table 4). As shown in Table 6, the SVM performs 9.1% better than StarryNite when tested on the new validation set. Furthermore, the similarity between the first and the second columns implies that the test data and the training data come from similar sources.

**Table 6:** Comparison of SVM and StarryNite on the new validation set.

Metric	CV SVM2	Dev SVM2	StarryNite
True Positives	4306	4571	4874
True Negatives	1273	902	0
False Positives	675	1046	1948
False Negatives	568	303	0
Sensitivity	88.34	93.78	100.0
PPV	86.45	81.38	69.39
Accuracy	81.78	80.23	71.13

Two sets of SVM results are included: from a collection of SVMs trained via cross-validation on the new validation data set ("CV SVM2"), and from a single SVM trained on the new development set ("Dev SVM2"), which is the combination of the initial development and validation sets

Note that in Table 6, the accuracies of both StarryNite and the SVM are lower than the results presented in Tables 2 and 5. This is mainly because all the images in our first dataset are sampled with 1 minute time resolution while some of the series in the second dataset have 1.5 minute resolution (see Methods for details of datasets). We also trained and tested our SVM classifier only on series with 1.5 minute resolution and obtained a similar drop in performance (data not shown). This result can be explained as follows. When the time resolution increases from 1 min to 1.5 min, the newborn cells that are sampled by the imaging protocol move further away from the parent cell, which makes it more difficult to detect divisions because right after a division we expect the parent and the children to be close to each other to some extent. Having a larger separation between parent and children cells leads to an increase in divisions detected as movements. On the other hand, because the newborn cells come closer to other cells that are actually moving, the number of movements detected as divisions increases. Therefore, having experiments with 1.5 min time resolution in our test set makes the classification task more challenging for both methods. Nonetheless, the performance of the SVM was significantly better than StarryNite, validating the success of our approach.

The final trained SVM, which is incorporated into the StarryNite program, is trained from all three data sets, in an effort to provide the best generalization performance.

## Discussion

To date, several experimental protocols have been developed to monitor differential gene expression during embryonic development in which image analysis plays a fundamental role for efficient recognition and annotation of cells [7-16]. To correctly identify and classify cells, these methods employ a collection of features, such as shape, geometry, texture, wavelet and moment features [3,12,13]. Often, feature reduction techniques are applied to the computed features (*e.g*. principal component analysis, linear discriminant analysis, maximum margin criterion, stepwise discriminant analysis based feature selection and genetic algorithm based feature selection) to achieve greater discriminative power [4,13,15,17].

In this work, instead of using features described by other research groups, we focused on relatively simple, biologically motivated features that have the potential to discriminate dividing cells from moving ones. There are a couple of reasons for this preference. First, the classification task we are solving is relatively straightforward compared with, for example, recognizing the individual phases of mitosis. Second, having a large feature set does not always guarantee improved recognition performance [12,13], even in the presence of sufficient training data. This is mainly because many features share the same information. In this paper, we also did not apply commonly used feature reduction techniques because, first, we have enough training data and, second, our preliminary feature analysis experiments suggest that feature selection is unlikely to lead to dramatically improved performance on this set of morphological features. On the other hand, the extension of the feature set to include other feature types is a possible direction. For instance, depending on the source of the image data, it may often happen that additional cell features might also be exploited that are specific to the tissue type or species. These features could be introduced to add even stronger support to automated error detection. In that case, more sophisticated feature selection methods can be exploited to extract the most informative feature set for a more accurate classifier. We leave this extension as future work.

## Conclusions

In this work, we concentrated on one of the most common errors made by StarryNite, which is image analysis software for automatic recognition and tracing of developing *C. elegans *cells in a 3D imaging protocol. We have shown that the error rate for calling a nucleus as dividing can be significantly reduced by an SVM classifier. The original level of errors that must be identified and corrected in a new StarryNite dataset constitutes approximately 12-30% of the original observations. Automated correction using the SVM then can be expected to reduce this error rate by 2-9%. This cuts the amount of manual corrective work by 16-30%. Our method may over-predict potential annotation errors in some cases, but those false predictions can actually be helpful in drawing attention to trouble spots for final, manual data cleanup, and thus speed that process.

This work suggests several avenues for future research. Perhaps most obviously, this post-processing approach can also be applied to other types of errors. It is also possible to relate one type of an error to another, because errors are frequently coupled. For instance, movement detected as division error can be coupled with division detected as movement. Similarly, a movement detected as cell death can be coupled with a movement detected as division at a later time point. In the future, by considering correlations among error types, it should be possible to design more accurate classifers. Alternatively, the decision mechanism of the SVM can be used in the context of the StarryNite algorithm, rather than as a post-processing step. Furthermore, it should be possible to utilize the topology of the *C. elegans *cell lineage tree during the annotation process. Though the exact time instances of divisions might vary from one embryo to another, the topology of the tree and the average division times are known *a priori*. Such information could be exploited by a more elaborate statistical model. Finally, the use of the SVM classifier is not limited to StarryNite only. It can easily be applied to other data types or utilized in projects in which a methodological error correction step is essential due to large data size. Therefore, our method offers a guide to a wider audience on how to test and assess such algorithms in general. Further advances in accurate annotations will make it possible to analyze gene expression in the later cell stages, which is important because the majority of genes responsible for embryonic development start differentiating after the 195 cell stage.

## Methods

### Benchmark datasets

This study relies upon two primary datasets. The first one contains a total of 20 small benchmark experiments, each edited up to the 195 cell stage. The second dataset contains 10 benchmark series, some of which are edited up to the 350 cell stage. For each experiment, we have annotations produced by StarryNite as well as the manually edited annotations, which we take as the ground truth in our simulations. Properties of the data sets are summarized in Tables 3 and 4.

We divided the first dataset into two groups of ten experiments: the development set and the validation set. We used the development data set to perform the individual feature analyses described under "Preliminary Feature Analysis" and "Feature Selection" and the validation set for reporting the accuracy.

To enrich our performance evaluation, we trained the SVM on the first dataset and computed the predictions and accuracy measures on the second dataset. In our simulations with the second dataset, we only considered data up to the 195 cell stage, to be consistent with the first dataset.

Because we concentrated on divisions annotated as movements, from each experiment series, we only extracted the division annotations generated by StarryNite. For simplicity, we eliminated the cases where StarryNite correctly detects a division but assigns the wrong nuclei as the children (*i.e*., indexing errors of division shown in Figure 2(b)). In each row of Tables 3 and 4, we show the number of correct division calls by StarryNite, incorrect division calls (which are actually movements), and the total number of selected nuclei in that series.

### Feature definitions

#### Age

To compute the age of a parent, we trace the parent backwards in time until we reach another division or the starting time point. Then the age of that parent is defined as the time elapsed between those two time indices. Computation of the ages is similar for the children. Instead of tracing a nucleus backwards in time, we trace it in the forward time direction until we reach a cell division, cell death or the last time point in the annotation data. Then the age of a child is computed as the time elapsed between those two time indices.

#### Distances

**Figure 7:**
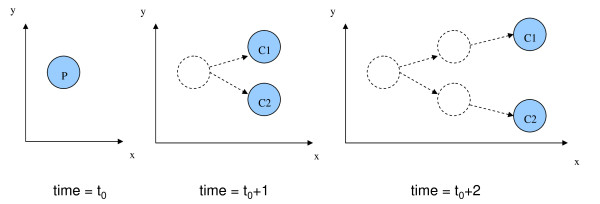
**Cell division followed by cell movements**. Parent nucleus at time = *t*_0 _divides into two children nuclei at *t*_0 _+ 1. Then the children move during the time elapsed between *t*_0 _+ 1 and *t*_0 _+ 2.

Let the time point at which we last observe the parent nucleus be *t*_0_. This means that at *t*_0 _+ 1 the parent nucleus is replaced with two children nuclei, as illustrated in Figure 7. We compute the following features: the distances in microns between the parent and its children (2 features), the distance between the two children at *t*_0 _+ 1 (1 feature), the distance between the children at *t*_0 _+ 2 (1 feature), distances of movement for the two children from *t*_0 _+ 1 to *t*_0 _+ 2 (2 features), distances between the nearest neighbors of the parent and the children (6 features), and distances between the parent and the nearest neighbors of the parent (3 features).

If a parent has fewer than three neighbors, then we omit that parent from our training data. For prediction, if a parent has fewer than three neighbors, we replace the features that requre the computation of nearest neighbors with their median values.

For consistency, we label the child that is closer to the parent nucleus as child-1 and the farther one as child-2. We then compute distance features accordingly (*i.e*., distance from child-1 to the nearest neighbor of the parent, etc).

#### Normalized nucleus support

In 3D time lapse microscopy imaging, each nucleus occupies a volume in three-dimensional space. Because we have image slices in the z direction, the image of a nucleus is contained in a set of x-y planes (typically 4 to 11 planes). The normalized nucleus support at the *i*^*th *^plane is defined as(1)

where *N*_*p *_is the number of pixels that have intensity greater than a threshold τ in a region of support denoted by ℛ, and *N *is the total number of pixels in that region. We define τ as(2)

where *I *is the image pixel intensity function. As the support region ℛ, we chose a square that has the same xy coordinates as the centroid of the nucleus and that has a side length equal to the diameter of the nucleus. We choose a square instead of a circle because we would like to be tolerant to the errors made by StarryNite in estimating the diameter. For instance, if the diameter of a nucleus is significantly underestimated, then the nucleus support would be computed as 1.0 both for a dividing and a non-dividing nucleus.

**Figure 8:**
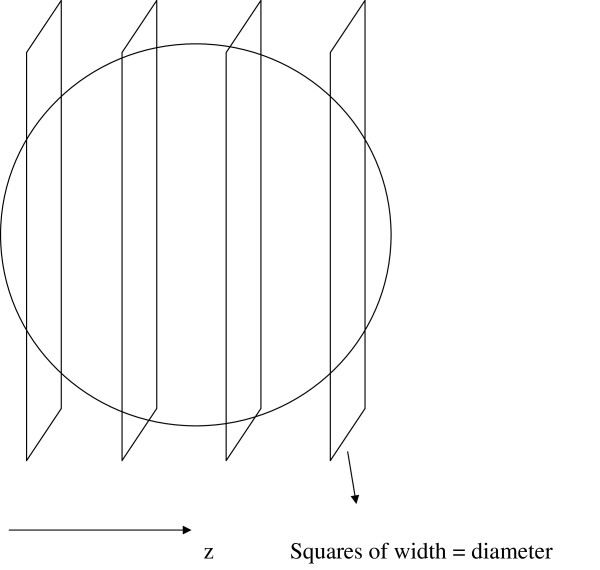
**Computation of the normalized nucleus support features**. A nucleus and the square regions on which the normalized nucleus supports are computed. Only 4 planes are shown for simplicity.

Because each nucleus can be represented by up to 11 planes in the z direction, and because image planes are 1 micron apart from each other, we computed the nucleus support at the plane that is closest to the centroid of the nucleus and at five planes on each side of the centroid, for a total of 11 features per nucleus (see Figure 8). We compute the normalized nucleus support features for the parent and the two children, yielding a total of 33 features. In addition, we compute the average and the standard deviation of these features separately for the parent and the two children, yielding six additional features.

#### Angle

Let (*x*_*P*_, *y*_*P*_, *z*_*P*_)^⊤ ^denote the 3 × 1 vector that contains the coordinate information for the centroid of the parent. Similarly let (*x*_*C*1_, *y*_*C*1_, *z*_*C*1_)^⊤ ^and (*x*_*C*2_, *y*_*C*2_, *z*_*C*2_)^⊤ ^denote the vectors that contain the coordinates for child-1 and child-2, respectively. Then the vector from the parent to child-1 becomes *u *= (*x*_*C*1 _- *x*_*P*_, *y*_*C*1 _- *y*_*P*_, *z*_*C*1 _- *z*_*P*_). Similarly, the vector from the parent to child-2 is computed as *v *= (*x*_*C*2 _- *x*_*P*_, *y*_*C*2 _- *y*_*P*_, *z*_*C*2 _- *z*_*P*_). The cosine of the angle between these vectors is(3)

where ||*u*|| is the length of *u *computed as (*u*^⊤^*u*)^1/2^.

We compute the cosines of the angles between the following vector pairs:

1. parent to child-1, parent to child-2

2. parent at *t*-1 to parent, parent to child-1

3. parent at *t*-1 to parent, parent to child-2

4. parent to child-1, child-1 to child-1 at *t *+ 2

5. parent to child-2, child-2 to child-2 at *t *+ 2

If we denote the time index of the parent by *t*, then the we are assuming that it moved from *t *- 1 to t. Similarly, the time indices of the children start from *t *+ 1. The first angle measures whether the two children move in opposite directions. The second and the third angles measure whether the separation of children is symmetric with respect to the parent's axis of movement from *t *- 1 to *t*. The fourth and fifth angles measure whether the children change their direction when moving from *t *+ 1 to *t *+ 2. We normally expect them to keep moving in the same direction unless there is a collision with other nuclei.

### SVM classifier

We implemented an SVM classifier [18,19] that operates on the division calls of StarryNite and decides whether a division annotation is correct or incorrect. We implemented our classifier using LIBSVM [20]. Before processing, each feature was linearly rescaled to the range [-1, 1]. For each cross-validation fold, we performed 5-fold internal cross-validation within the training set, selecting two hyperparameters, the regularization parameter *C *∈, {2^-5^, 2^-3^, ..., 2^15} ^and the radial basis function kernel width γ ∈, {2^-15^, 2^-13^, ..., 2^3} ^to maximize accuracy. Subsequently, the selected hyperparameters were used to train an SVM on the entire training set, and the learned SVM was applied to the test set.

## Authors' contributions

WSN, JIM and RHW coordinated the study. ZA, JM and WSN developed features. JIM and RHW provided the datasets. ZA implemented the algorithm and evaluated its performance. ZA and WSN wrote the manuscript. All authors read and approved the final manuscript.

## Supplementary Material

Additional file 1 **Features, feature groups and AUC scores**. The set of 82 features that are originally considered for the SVM classifier. The feature names and the group numbers are tabulated. Features are sorted with respect to their AUC scores and the ones that have zero AUC scores are then excluded from the classifier. Click here for file
